# Survival and analysis of prognostic factors for severe burn patients with inhalation injury: based on the respiratory SOFA score

**DOI:** 10.1186/s12873-022-00767-6

**Published:** 2023-01-05

**Authors:** Qiang Ji, Jun Tang, Shulian Li, Junjie Chen

**Affiliations:** 1 Department of Burn and Plastic Surgery, West China Hospital, Sichuan University, Guoxue Alley, Wuhou District, 610041 Chengdu, China; 2 Department of Thyroid Surgery, West China Hospital, Sichuan University, Guoxue Alley, Wuhou District, 610041 Chengdu, China

**Keywords:** Severe burn, Inhalation injury, SOFA score, Survival analysis

## Abstract

**Background:**

It is important to determine the severity of inhalation injury in severely burned patients. The oxygenation index PaO_2_/FiO_2_(PF) ratio is a key clinical indicator of inhalation injury. Sequential organ failure assessment (SOFA) is developed to assess the acute incidence of critical illness in the population. We hope to provide an assessment of survival or prognostic factor for severely burned patients with inhalation injury based on the respiratory SOFA score.

**Methods:**

This is a retrospective cohort study of all admissions to Department of Burn and Plastic Surgery at West China Hospital of Sichuan University from July 2010 to March 2021. Data was analyzed using Cox regression models to determine significant predictors of mortality. Survival analysis with time to death event was performed using the Kaplan–Meier survival curve with the log-rank test. All potential risk factors were considered independent variables, while survival was considered the risk dependent variable.

**Results:**

One hundred eighteen severe burn patients with inhalation injury who met the inclusion and exclusion criteria were admitted, including men accounted for 76.3%. The mean age and length of stay were 45.9 (14.8) years and 44.3 (38.4) days. Flame burns are the main etiology of burn (74.6%). Patients with the respiratory SOFA score greater than 2 have undergone mechanical ventilation. Univariate Kaplan–Meier analysis identified age, total body surface area burned (TBSA), ICU admission and the respiratory SOFA score as significant factors on survival. Cox regression analysis showed that TBSA and the respiratory SOFA score were associated with patient survival (*p* < 0.001). In some patients with severe burns and inhalation damage, the survival probability drops to less than 10% (TBSA greater than 80%: 8.9% and respiratory SOFA score greater than 2: 5.6%). This study statistically found that the TBSA with the respiratory SOFA score model (AUROC: 0.955) and the rBaux score (AUROC: 0.927) had similar predictive value (*p* = 0.175).

**Conclusion:**

The study indicates that a high respiratory system SOFA score was identified as a strong and independent predictor of severely burned patients with inhalation injury during hospitalization. When combined with TBSA, the respiratory SOFA scores can dynamically assess the severity of the patient's lung injury and improve the predictive level.

**Supplementary Information:**

The online version contains supplementary material available at 10.1186/s12873-022-00767-6.

## Introduction

Large-area burns combined with inhalation injury may make patients more susceptible to infection, increase the risk of death, and lengthen hospital stays [1–3]. According to data, 30% of burn patients suffer from inhalation injury, and the mortality and morbidity rates associated with burns have risen from 3 to 10% and 20% to 30%. [4]. The revised Baux score (rBaux) is regarded as an excellent scoring system for predicting burn mortality, with three parameters (age, %TBSA and inhalation injury) [5, 6]. Although many studies have shown that the increasing inhalation injury only slightly improves the ability to predict mortality, it is simple to increase the Baux score by 17 points, and there is no dynamic scoring system to assess the severity of airway and lung injury during the patient's hospitalization [7].

The SOFA (Sequential Organ Failure Assessment) score involving 6 body systems was developed by European Society of Intensive Care Medicine in 1994, the aim of which was to create a standard to describe the degree of single organ dysfunction/failure in as objective, simple and continuous a form as possible [8]. The SOFA score has been widely regarded as an important grading method for predicting sepsis mortality [9]. Narvaez et al. demonstrated that the SOFA score is the only clinical factor that can identify survivors of community-acquired pneumonia with acute respiratory distress syndrome (ARDS) after comparing multiple severity assessment scores [10]. The score is the same as the APACHE score (Acute Physiology and Chronic Health Evaluation), which can be repeatedly calculated to evaluate the organ dysfunction of critically ill patients during the treatment process.

The respiratory SOFA score is based on the PaO_2_/FiO_2_ (PF) ratio and whether associated with mechanical ventilation, and ranges from 0 to 4 points with an increasing score reflecting worsening respiratory dysfunction [11]. Inhalation injury is usually accompanied by a decrease in the PF ratio, which results in pathophysiological alterations in the lung parenchyma, as well as hypoxia [12]. A number of studies have also indicated that the PF ratio is a key predictor of inhalation damage patients' prognosis. A study found that PF levels are highly correlated with smoke inhalation injury in burned children [13]. Walsh et al. showed that the airway microbiota following burn and inhalation injury with a PF ratio ≤ 300 mmHg was altered [14]. Sekulic et al. found that in mechanically ventilated patients, SOFA scores measured on the third and seventh days have important predictive significance for the prognosis of critically ill hospitalized patients in ICU [15]. At present, there have been few studies on the value of the respiratory SOFA score in the prognosis of severe burn patients with inhalation injury. So, the authors put forward a hypothesis that the respiratory SOFA score also maybe be an important prognostic factor, and then decided to conduct a retrospective study to determine whether it is relevant to the survivals of patients.

## Materials and methods

### Collection of baseline information

This study was granted approval by the West China ethics committee of Sichuan University. The institutional review board granted permission to access and use the medical records and waived the need for informed consent for this retrospective analysis. Based on the diagnostic criteria for inhalation burns, the retrospective analysis method was used to design a survey form for severe burn patients with inhalation injury who had been admitted in the Department of Burn and Plastic Surgery of West China Hospital of Sichuan University from July 2010 to March 2021. All patients were confirmed negative report on COVID-19 RT-PCR test. The clinical data and Serum physiological indicators were collected at 6 different time points (on admission and every 12 h) within 72 h after the onset of the burn, and the worst results were taken. The initial intravenous infusion volume is calculated by 2 mL/kg/1%TBSA established by the Burn Department of West China Hospital of Sichuan University within the first 72 h, based on 2:1 or 1:1 ratio of crystal and colloid solution, and glucose injection was added to maintain adequate urine volume. Tracheotomy for invasive continuous positive airway pressure was performed in all mechanically ventilated patients, or positive end-expiratory pressure (PEEP) and tidal pressure were determined by pressure–volume curves and tidal volume: 6-8 ml/Kg. All patients' wounds were covered with silver sulfadiazine and mupirocin ointment. Patients with severe and extensive burns or with annular eschar in the case of the thorax and extremities underwent escharotomy for decompression procedures, when the respiratory function and the circulation are impaired. Burn patients requiring mechanical ventilation, or with symptoms such as acute kidney injury and sepsis will be admitted to the ICU. Patients with mild inhalation injury only inhaled low-flow oxygen, and moderate or severe patients inhaled mucosolvan injection, and some patients used budesonide to control airway inflammation.

### Study design and diagnostic criteria

#### Inclusion criteria

(1) all patients with inhalation injury were diagnosed by bronchoscopy. (2) the patients with a history of smoke exposure in a closed environment. (3) patients accompanied by severe head and face burns, or formation of annular and semi annular eschar. (4) patients older than 18 years were included. (5) burn area was greater than 20% were included in the study.

#### Exclusion criteria

(1) patients with a history of burn-surgery performed in other hospitals. (2) patients with a history of respiratory disease or systemic inflammatory response syndrome. (3) patients were accompanied by multiple injuries in other body systems, (4) patients who stopped treatment during hospitalization or were discharged automatically after receiving treatment.

The respiratory SOFA scores were assigned based on the following PaO_2_/FiO_2_ (PF) ratios: ≥ 400 mmHg, 0 point; 301–399 mmHg, 1 point; 200–300 mmHg, 2 point; 100–199 mmHg + mechanical ventilation (noninvasive/invasive), 3 point; and < 100 mmHg + mechanical ventilation (noninvasive/invasive), 4 point.

### Statistical analysis

The mean ± standard deviation was used to express measurement data with a normal distribution. A single factor analysis was performed using an unpaired data t-test; the measurement data did not have a normal distribution, with the mean falling between Q1 and Q3. The mean values obtained using Pearson’s χ^2^ test were used in the univariate analysis; indices with *P* < 0.05 were used as independent variables, while the proportion of severe burn patients with inhalation injury who died was used as the dependent variable. Survival time was estimated from the date of injury to either death (if died) or date of discharge. Kaplan–Meier plots were used to display the survival probability of the different grades at different times and a log-rank test was used to compare between group survival times. Multivariate Cox’s regression analysis was performed, and variables with *P* < 0.05 were included in the regression analysis to determine the risk factors of severe burn with inhalation injury. All statistical analyses were performed using SPSS 22.0 statistical software and the R statistical software (version 3.6.1) to calculate the sensitivity, specificity, and accuracy of the area under the ROC curve. All methods were performed in accordance with the relevant guidelines and regulations.

## Results

### Comparison of clinical data of patients and burn-specific severity scores

As shown in Table 1, the factors considered to predict mortality during the hospital stay included age, sex, smoking, etiology of burn, surgery, tracheotomy, ICU admission, and length of hospital stay, TBSA, and respiratory SOFA score. Patients with inhalation injury who satisfied the inclusion and exclusion criteria were hospitalized, with men accounting for 76.3 percent of those admitted. The average age and length of stay were 45.9 (14.8) years and 44.3 (38.4) days, respectively. Flame burns are the most common cause of burns (74.6%). Among them, 84 individuals survived, while 34 died while in the hospital. The median TBSA percentage was 54.0%. 17.8% of burn patients completed escharectomy surgery within 72 h, and 58.5% underwent tracheotomy. Tweenty-one patients were admitted to the ICU. The number of individuals having a respiratory SOFA score of 3–4 was 19 (16.1%).

**Table 1:** Patient demographics and comparison between survivors and non-survivors

	**Survivors**	**Non-survivors**	**Total**
	**(*N* = 84)**	**(*N* = 34)**	**(*N* = 118)**
**Sex**			
Male	64 (76.2%)	26 (76.5%)	90 (76.3%)
Female	20 (23.8%)	8 (23.5%)	28 (23.7%)
**Age (years)**			
Mean (SD)	42.9 (13.1)	53.1 (16.2)	45.9 (14.8)
Median [Min, Max]	44.5 [18.0, 69.0]	48.5 [32.0, 98.0]	45.0 [18.0, 98.0]
**Smoking**			
No	53 (63.1%)	19 (55.8%)	72(61.0%)
Yes	31 (36.9%)	15 (44.2%)	46(39.0%)
**Etiology of burn**			
Flame	59 (70.2%)	29 (85.3%)	88 (74.6%)
Scald	19 (22.6%)	4 (11.8%)	23 (19.5%)
Flash	6 (7.1%)	1 (2.9%)	7 (5.9%)
**TBSA**			
Mean (SD)	47.1 (16.6)	79.5 (17.2)	56.4 (22.3)
Median [Min, Max]	45.5 [20.0, 85.0]	84.0 [40.0, 100]	54.0 [20.0, 100]
**Surgery**			
No	72(85.7%)	25(73.5%)	97(82.2%)
Yes	12(14.3%)	9(26.5%)	21(17.8%)
**Tracheotomy**			
No	37 (44.0%)	12 (35.3%)	49 (41.5%)
Yes	47 (56.0%)	22 (64.7%)	69 (58.5%)
**ICU admission**			
No	75 (89.3%)	22 (64.7%)	97 (82.2%)
Yes	9 (10.7%)	12 (35.3%)	21 (17.8%)
**LOS**			
Mean (SD)	56.8 (37.9)	13.2 (15.2)	44.3 (38.4)
Median [Min, Max]	48.0 [1.00, 189]	5.50 [1.00, 56.0]	39.0 [1.00, 189]
**Respiratory SOFA score**			
0	16 (19.0%)	2 (5.9%)	18 (15.3%)
1	51 (60.7%)	3 (8.8%)	54 (45.8%)
2	15 (17.9%)	12 (35.3%)	27 (22.9%)
3	2 (2.4%)	14 (41.2%)	16 (13.6%)
4	0 (0%)	3 (8.8%)	3 (2.5%)

### Kaplan–Meier survival analysis and log rank test

**Fig. 1:**
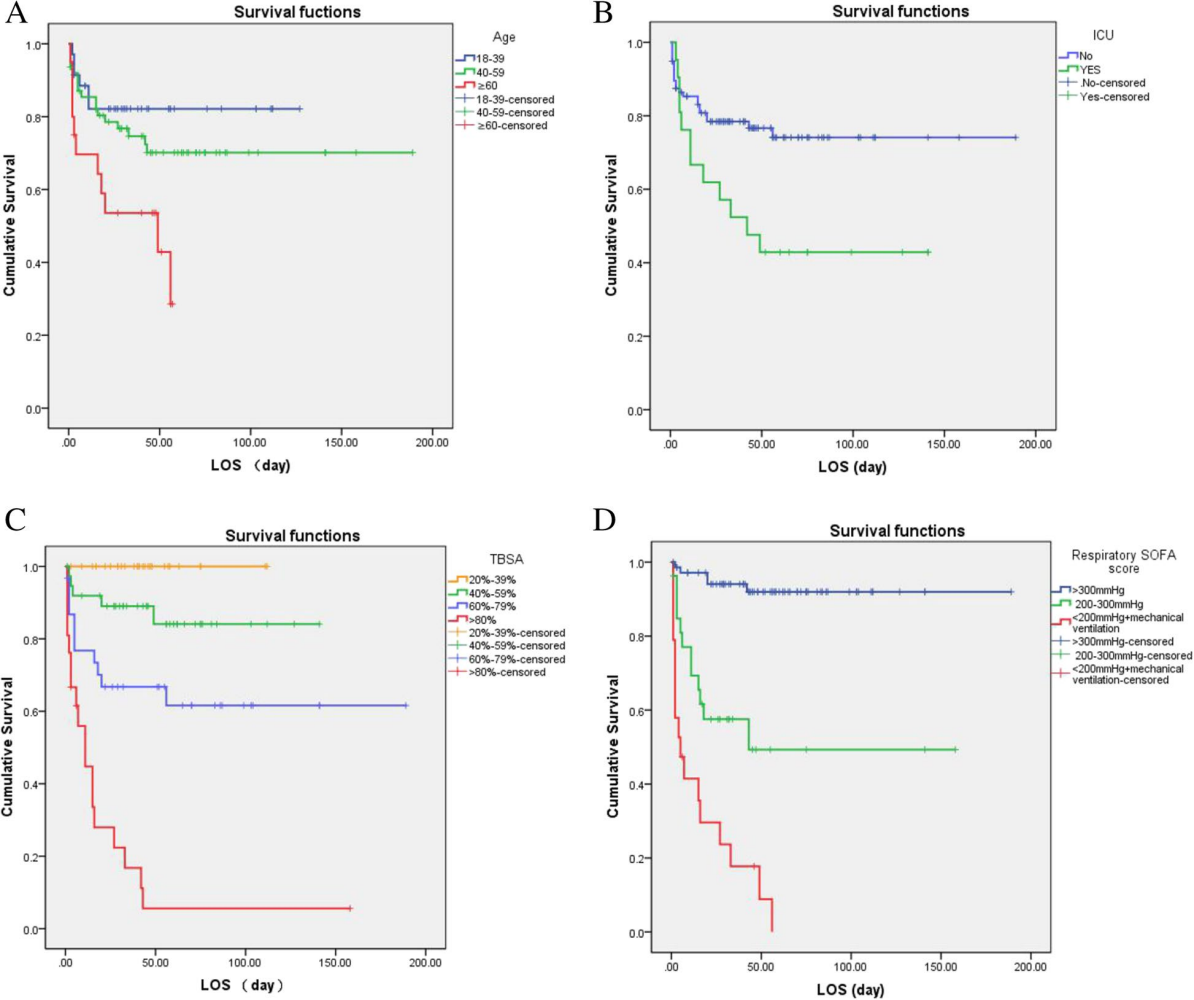
Kaplen-Meier survival curve of significant factors of burn mortality. **A** Age. **B** ICU admission. **C** Total body surface area (TBSA) > 20%. **D** the respiratory SOFA score (The blue drawn curve for 0–1 points, PF > 300 mmHg; The green drawn curve for 2 point, PF = 200–300 mmHg; The red drawn curve for 3–4 points, PF < 200 mmHg + mechanical ventilation)

As shown in the Table 2, age (log rank *p* = 0.010), ICU admission (log rank *p* = 0.009), TBSA (log rank *p* < 0.001), and the respiratory SOFA score (log rank *p* < 0.001) were all substantially linked with shorter time to death by univariate Kaplan–Meier survival analysis. Statistics show that the elderly, ICU admission, large-area TBSA, and respiratory SOFA scores of 3–4 all revealed an increased risk of death. Using the respiratory SOFA score, however, our findings revealed a lower probability of survival in severe burns patients with inhalation injury, when the survival probability drops to less than 10%. (TBSA more than 80 percent: 8.9% and respiratory SOFA score greater than 2: 5.6%). Patients with more than 80% TBSA had a median survival time of 11 days. The mean survival time with a respiratory SOFA score larger than 2 (PaO_2_/FiO_2_: < 200 mmHg + mechanical ventilation) was substantially shorter than with a score of 0–1 (PaO_2_/FiO_2_: 200-300 mmHg and > 300 mmHg). The previous median survival time was associated with a shorter hospital stay (5 days) (Fig. 1).

**Table 2:** Univariate analysis for predictors associated with mortality

	*χ*^*2*^	*P*-value
Sex(Male vs Female)	0.111	0.739
Age (18–39, 40–59 vs > 60 years)	9.297	0.010
Smoking (No vs Yes)	0.529	0.534
Etiology of burn (Flame, Scald vs Flash)	59	0.232
TBSA (20%-39%, 40%-59% 60%-79% vs > 80%)	68.115	< 0.001
Surgery (No vs Yes)	2.456	0.182
Tracheotomy (No vs Yes)	0.432	0.511
ICU admission (No vs Yes)	6.765	0.009
Respiratory SOFA score (0–1 points, 2 points vs 3–4 points)	79.082	< 0.001

### Multivariate Cox risk regression

**Fig. 2:**
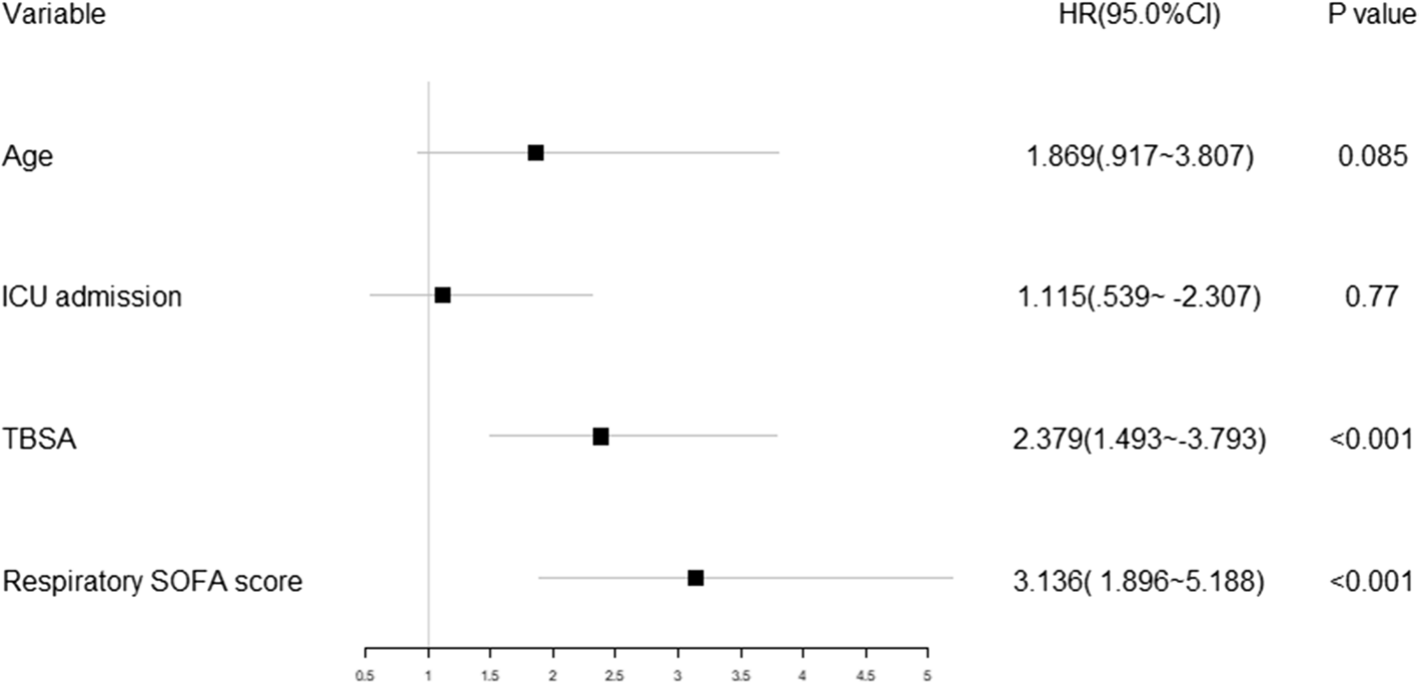
the results of the forest plot with analysis of Cox regression models

In multivariate Cox regression analysis, however, the study found that TBSA (HR = 2.379, 95% CI 1.493–3.793, *p* < 0.001) and the respiratory SOFA score (HR = 3.136, 95% CI 1.896–5.188, *p* < 0.001) were independently associated with shorter time to death (Fig. 2).

### Analysis of the ROC curve

**Fig. 3:**
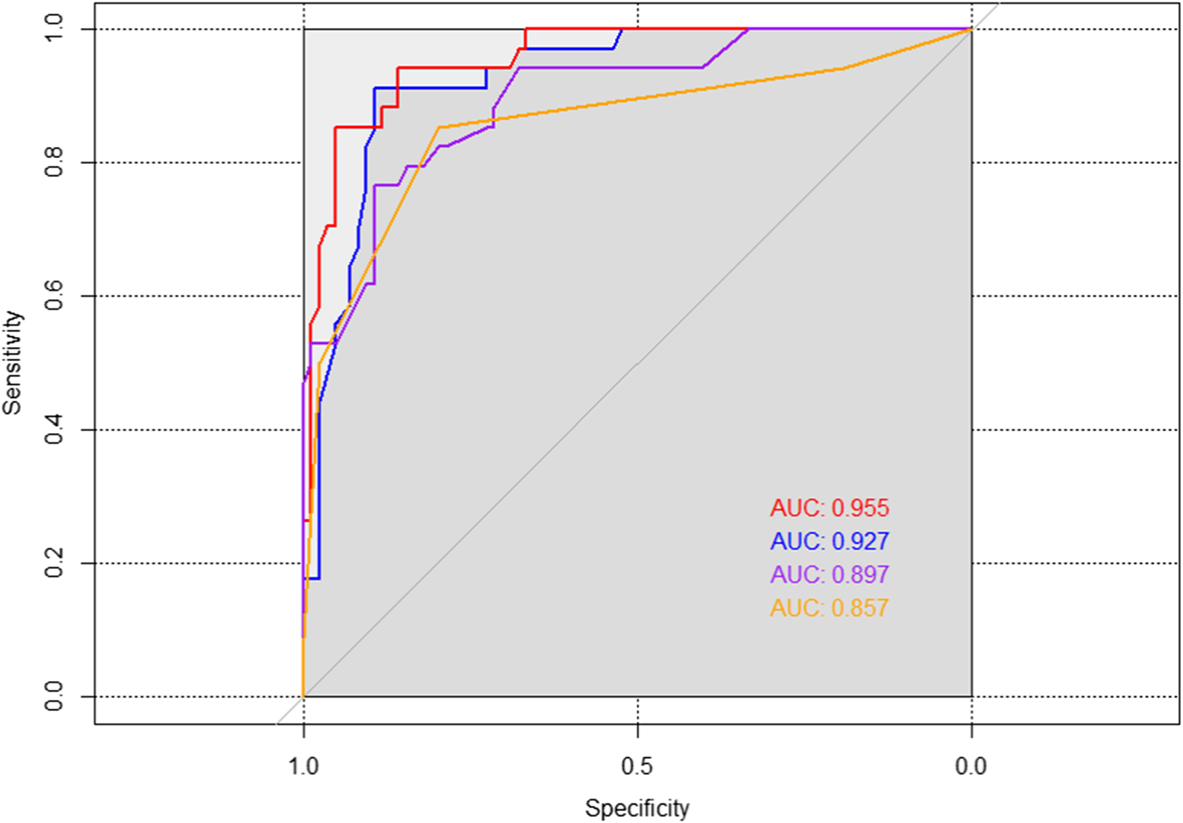
Analysis of the ROC curve values: The purple drawn curve for TBSA; The orange drawn curve for the respiratory SOFA score; The red drawn curve for the respiratory SOFA scores combined with TBSA. The blue drawn curve for the rBaux score

Using a Hosmer–Lemeshow test and the total AUROC, we evaluated the differences in prediction under both risk variables. The curves showed that respiratory SOFA score and TBSA (AUROC: 0.857 and 0.897) were good predictors of death in severe burn patients with inhalation injury. This study statistically found that the TBSA with the respiratory SOFA score model (AUROC: 0.955) and the rBaux score (AUROC: 0.927) had similar predictive value (*p* = 0.175) and the continuous evaluation of the respiratory SOFA score made this model have the advantage of dynamically evaluating the prognosis of patients. The TBSA with the respiratory SOFA score accurately predicts the likelihood of negative outcomes in patients: a sensitivity of 0.952 and a specificity of 0.853. (In Supplementary 1 and Fig. 3).

## Discussion

Over a 10-year period, 118 severe burn patients with inhalation injury from the Department of Burns and Plastic Surgery, West China Hospital of Sichuan University, evaluated retrospectively. The study indicates that a high respiratory system SOFA score and large-area TBSA are risk factors impacting patient survival, with both having high predictive values. The combined evaluation level of the two factors reaches the diagnostic value of the rBaux score.

In this survival study's Kaplan–Meier analysis, the inpatient survival time of elderly patients over 60 years was considerably less than that of other patients. The exact cause might be related to thinner skin, diminished feeling, mental changes, pre-existing comorbidities, or a variety of other circumstances [16, 17]. Although the Cox multivariate analysis outcomes do not include the element of age, we cannot entirely rule out this risk factor from the revised Baux score for burns. In our study, older age had a nearly significant *p* value of 0.05 (*p* = 0.085), indicating a substantial link with death. According to logistic regression analysis, Henry et al. also reported that older age is not a mortality predictor of burn injury due to the small sample population of elderly patients [18]. Inhalation injury frequently aggravates the disease, requiring some patients to be moved to the ICU ward for respiratory support therapy, reducing their hospital survival time. However, ICU hospitalization variables, on the other hand, are not predictors of patient survival. Severe burns can impede skin function substantially, produce significant fluid loss, harm the interior environment, and raise the risk of infection [19–21]. Extensive TBSA burns are still an important risk factor for patients in our research, which is consistent with the majority of prior investigations.

Some studies suggest that when evaluating the application of the oxygenation index score in burn patients with inhalation injury, the PF ratio cannot indicate the severity of inhalation injury, and mechanical ventilation should be considered a risk factor for mortality [22, 23]. Furthermore, when evaluating patients with inhalation injury, adding 17 points to the Baux score might easily lead to clinicians underestimating the impact of inhalation damage on patients' survival chances, and the rBaux score lacks the clinical indicators of dynamic evaluation. inhalation injury. These patients are frequently accompanied with a certain degree of secondary pneumonia in the early stages of burns, and have a greater mortality and comorbidities than patients without inhalation injury [24]. Many studies have also created other scoring systems for predicting the prognosis of burn patients; however, when utilized therapeutically for patients with inhalation injury, these scoring systems fail to take into account pathophysiological changes in pneumonia, carbon monoxide levels, and oxygenation levels [25–28]. The acute respiratory distress syndrome (ARDS) is the major cause of mortality in individuals with inhalation injury [29–31]. There are also various standards for detecting ARDS, such as the systemic inflammatory response syndrome (SIRS) and Berlin standards [32]. However, reliable evaluation of the independent respiratory system in severe burn patients with inhalation injury remains a challenge.

According to the revised Sepsis-3 (2016) consensus guideline, the SOFA scores predict the outcome of critically sick patients better than SIRS that characterizes sepsis. Although quickly SOFA (qSOFA), a relatively simple and affordable bedside clinical score, is thought to have a lesser predictive value than the revised Baux score, the usefulness of SOFA in assessing multiple systems functions in critically sick patients cannot be overstated. However, the SOFA score is especially useful for complete dynamic grading. Based on the patient's baseline risk level, it is currently believed that a SOFA score of 2 or higher indicates increasing by 2 to 25 times in the risk of death when compared to patients with a SOFA score of less than 2 [33, 34]. Moreover, the respiratory SOFA scores of 3–4 includes mechanical ventilation components. As a result, we divided the study population into three groups based on the above-mentioned SOFA score reasons. The results showed that the respiratory SOFA scores can be a risk factor for survival in individuals with severe burns and inhalation injuries. A 12-year retrospective research undertaken by Swanson and their colleagues found that lung damage is the second most prevalent cause of mortality in the first week following burns (84%), after only burn shock (62%) [35]. Our findings are consistent with this result. It was discovered that the median survival period of patients with a respiratory system SOFA score more than 2 was only 5 days, and the survival rate was only 79%, showing that the more serious the inhalation injury, the higher the patient's likelihood of dying. Further investigation revealed that the respiratory SOFA score has a comparable predictive value to the TBSA (AUROC: 0.857 and 0897). When the two clinical indicators are combined to assess the prognosis of patients, they have similar value to the rBaux score and can dynamically assess and monitor patient prognosis.

There are some limitations to this study that must be addressed. First, the study was designed using data from a small sample size obtained from a single center, which did not fully account for all potential influencing factors; second, because this was a retrospective review, there may have been some selection biases and errors in record entry; thus, prospective studies with a large sample size from multiple institutions are required; and third, severe burn with inhalation injury in the pediatric population was not included in this study.

## Conclusion

In conclusion, this study retrospectively analyzed the admission data of 118 severe burn patients with inhalation injury (> 20% TBSA) who were treated at a burn center in southwest China. The findings indicate that a high respiratory system SOFA score was identified as a strong and independent predictor during hospitalization during the early phase. When combined with TBSA, the SOFA assessment of the respiratory system can dynamically assess the severity of the patient's lungs, improving the predictive level of patients with inhalation burns.

## Supplementary Information

**Additional file 1:** **Supplementary material 1. **Receiver operating characteristic curve diagnostic value for prognostic factors for severe burn patients with inhalation injury.

## Data Availability

The datasets used and analysed during the current study are available from the corresponding author on reasonable request.
