# Supplementary material for: Survival and analysis of prognostic factors for severe burn patients with inhalation injury: based on the respiratory SOFA score
Source: BMC Emerg Med. 2023 Jan 5;23:1. doi: 10.1186/s12873-022-00767-6 (PMC9813898; doi:10.1186/s12873-022-00767-6)
Supplement: Supplementary file 1 — Additional file 1: Supplementary material 1. Receiver operating characteristic curve diagnostic value for prognostic factors for severe burn patients with inhalation injury. [file 12873_2022_767_MOESM1_ESM.docx]

**Supplementary material 1. Receiver operating characteristic curve diagnostic value for prognostic factors for severe burn patients with inhalation injury.**

| **Supplementary** 1- **Diagnostic value of the receiver operating characteristic curve** | | | | | |
| --- | --- | --- | --- | --- | --- |
| Predictor | AUC | Sensitivity. | Specificity | OR (95.0%*CI*) | *P* |
| TBSA | 0.897 | 0.892 | 0.765 | 3.349 (2.149-4.548) | 0.049 |
| the respiratory SOFA scores | 0.857 | 0.798 | 0.852 | 1.897 (1.203-2.590) | 0.004 |
| rBaux | 0.927 | 0.893 | 0.921 | 4.656 (2.947-6.365) | 0.175 |
| TBSA with the respiratory SOFA scores | 0.955 | 0.952 | 0.853 | ------------ | Reference |

AUC, area under the curve;
